# Measuring inequalities in COVID-19 vaccination uptake and intent: results from the Canadian Community Health Survey 2021

**DOI:** 10.1186/s12889-022-14090-z

**Published:** 2022-09-08

**Authors:** Mireille Guay, Aubrey Maquiling, Ruoke Chen, Valérie Lavergne, Donalyne-Joy Baysac, Audrey Racine, Eve Dubé, Shannon E. MacDonald, Nicolas L. Gilbert

**Affiliations:** 1 Vaccine Rollout Task Force, Public Health Agency of Canada, Ottawa, ON Canada; 2 Centre for Population Health Data, Statistics Canada, Ottawa, ON Canada; 3 Institut National de Santé Publique du Québec, Québec, QC Canada; 4 Département d’anthropologie, Université Laval, Québec, QC Canada; 5 Faculty of Nursing, University of Alberta, Alberta, Canada; 6 École de santé publique de l’Université de Montréal, Montréal, QC Canada

**Keywords:** COVID-19, Vaccine, Vaccination Coverage, Intention, Health equity, Canada

## Abstract

**Background:**

By July 2021, Canada had received enough COVID-19 vaccines to fully vaccinate every eligible Canadian. However, despite the availability of vaccines, some eligible individuals remain unvaccinated. Differences in vaccination uptake can be driven by health inequalities which have been exacerbated and amplified by the pandemic. This study aims to assess inequalities in COVID-19 vaccination uptake and intent in adults 18 years or older across Canada by identifying sociodemographic factors associated with non-vaccination and low vaccination intent using data drawn from the June to August 2021 Canadian Community Health Survey (CCHS).

**Methods:**

The CCHS is an annual cross-sectional and nationally representative survey conducted by Statistics Canada, which collects health-related information. Since September 2020, questions about the COVID-19 pandemic are asked. Adjusted logistic regression models were fitted to examine associations between vaccination uptake or intent and sociodemographic and health related variables. Region, age, gender, level of education, Indigenous status, visible minority status, perceived health status, and having a regular healthcare provider were considered as predictors, among other factors.

**Results:**

The analysis included 9,509 respondents. The proportion of unvaccinated was 11%. Non-vaccination was associated with less than university education (aOR up to 3.5, 95% CI 2.1–6.1), living with children under 12 years old (aOR 1.6, 95% CI 1.1–2.4), not having a regular healthcare provider (aOR 1.6, 95% CI 1.1–2.2), and poor self-perceived health (aOR 1.8, 95% CI 1.3–2.4).

Only 5% of the population had low intention to get vaccinated. Being unlikely to get vaccinated was associated with the Prairies region (aOR 2.2, 95% CI 1.2–4.1), younger age groups (aOR up to 4.0, 95% CI 1.3–12.3), less than university education (aOR up to 3.8, 95% CI 1.9–7.6), not being part of a visible minority group (aOR 3.0, 95% CI 1.4–6.4), living with children under 12 years old (aOR 1.8, 95% CI 1.1–2.9), unattached individuals (aOR 2.6, 95% CI 1.1–6.1), and poor self-perceived health (aOR 2.0, 95% CI 1.3–2.9).

**Conclusions:**

Disparities were observed in vaccination uptake and intent among various sociodemographic groups. Awareness of inequalities in COVID-19 vaccination uptake and intent is needed to determine the vaccination barriers to address in vaccination promotion strategies.

## Background

Canada initiated its COVID-19 vaccination campaign on December 14, 2020 to first administer doses to priority groups (i.e. those at high risk of severe illness and death from COVID-19 and those who are most likely to be exposed to the virus: residents and staff of congregate living settings, frontline healthcare workers, adults in Indigenous communities, adults of advanced age) and then followed with broader availability to the public throughout 2021. Vaccine prioritization varied by province, but was predominantly based on age, starting with the elderly and then decreasing in 5- or 10-year age bands. In the ten Canadian provinces, all adults 18 years or older became eligible to receive their first dose between May 10 and May 27, 2021. By the end of July, enough vaccine doses had been acquired to fully vaccinate every eligible person in Canada [1].

Health inequities, which have been exacerbated and amplified by the pandemic, are among the diverse forms of barriers as they affect the accessibility, acceptance and uptake of COVID-19 vaccines in equity-seeking groups [2, 3]. Health inequalities represent any measurable difference in health across individuals or socially relevant subpopulations [4]. When these differences are preventable, unjust or unnecessary, they are considered health inequities [4]. By measuring health inequalities, we can develop a better understanding of how to prevent them from persisting unfairly and further contributing to health inequities [4].

Health inequalities related to vaccination intent were observed in different groups in Canada. Indeed, younger age, lower education or income, identifying as non-white or Indigenous, being born outside of Canada and living in a household of at least five members were negatively associated with the intent to get vaccinated [5–7]. In the COVID-19 Vaccination Coverage Survey, males, and individuals with lower education or household income had higher odds of not intending to get vaccinated [8].

While the literature on vaccination intent provides insight on how sociodemographic factors influences health inequalities in vaccination uptake, few articles have yet to be published on disparities in vaccination uptake in Canada. Of the few studies available, most came from the US and the UK where findings indicated that vaccination coverage differ across gender, socioeconomic status, visible minority status and area of residency [9–11]. Despite having positive intentions to vaccinate, health inequalities in vaccination uptake can also occur in some groups or individuals due to systemic barriers in access [2].

As the pandemic continues to unfold, it is critical to ensure equitable distribution and uptake of COVID-19 vaccines in order to reduce morbidity and mortality. Understanding how socioeconomic inequalities have been impacting the current COVID-19 vaccination campaign in Canada is crucial to help public health authorities improve vaccine acceptance in order to limit hospitalizations and deaths, especially given the disproportionate health and economic impact COVID-19 has on marginalized groups [12]. In that regard, the annual Canadian Community Health Survey (CCHS), which collects health-related data, was leveraged by adding a COVID-19 module asking questions on vaccination uptake and intent. Using CCHS data, the purpose of this study was to assess inequalities in COVID-19 vaccination uptake and intent at the national level by identifying sociodemographic factors associated with non-vaccination and low vaccination intent.

## Methods

### Data source and study sample

The data used in this study were drawn from the 2021 collection cycle of the Canadian Community Health Survey. The CCHS is an annual cross-sectional and nationally representative survey conducted by Statistics Canada. Briefly, the survey collects information on health status, health care utilization and health determinants for Canadians 12 years or older residing in the 10 provinces and 3 territories, excluding full-time members of the Canadian Forces, children in foster care, those who live in institutions, those who live on reserve, as well as those living in Nunavik and Terres-Cries-de-la-Baie-James. The survey sample was selected using a multistage stratified cluster design. Due to the complex survey design, survey weights were given to each respondent and were calibrated by province, age group, and sex to ensure that the sample data is representative of the Canadian population. More details on the survey design and sampling method are available in a previously published report [13]. In September 2020, questions about experiences related to the pandemic along with a question on COVID-19 vaccination intent were introduced, followed by a question on COVID-19 vaccination uptake in March 2021.

The CCHS has multiple non-overlapping collection periods throughout the year and this study is based on the collection period for the months of June to August 2021. For this specific period, data was collected between June 1^st^ and September 5^th^ exclusively using computer-assisted telephone interviewing (CATI). The data file used for the current study included only respondents living in the provinces for a total of *n* = 10,093 participants. Since adolescents 12 to 17 years old were only offered appointments for COVID-19 vaccines towards the end of May in most provinces and would not have had sufficient time to get vaccinated prior to the start of the data collection period, they were excluded from this study resulting in a final sample of *n* = 9,509. The response rate for the population of study, Canadians aged 18 and older living in the provinces, was 22.6%.

### Measures

Respondents were asked to provide information on sociodemographic and health-related characteristics, as well as experiences during the COVID-19 pandemic.

#### Vaccination status

Participants were asked the following question: “Have you been vaccinated against COVID-19?” to which they could respond with “Yes, received at least one dose” or “No”. The responses from this question were used to represent vaccination status as one of two binary outcome variables.

#### Vaccination intent

For those who responded “No” to the previous question, a follow-up question was asked: “How likely is it that you would get a COVID-19 vaccine? Would you say…?” to which respondents answered using a 4-point Likert scale: ‘Very likely’, ‘Somewhat likely’, ‘Somewhat unlikely’, and ‘Very unlikely’. Responses were then regrouped to derive the second binary outcome variable, vaccination intent. Respondents who said they were vaccinated with at least one dose and those who were not vaccinated but who reported being very or somewhat likely to get a vaccine were recoded as being “vaccinated or likely to get vaccinated”. Those who reported being very or somewhat unlikely to get a vaccine were recoded as being “unlikely to get vaccinated”.

#### Sociodemographic factors

The CCHS collects information on several sociodemographic factors. For this study, predictors of interest were selected a priori based on factors previously demonstrated to be related to the modeled outcome according to literature and/or factors considered by the authors to conceptually have a potential association. Region of residence, age group, gender, level of education, Indigenous status, employment status, marital status, immigration status or country of birth, visible minority status, presence of children under 12 years old in the household, mother tongue, household composition, and dwelling ownership status were included as predictors. Data from Newfoundland and Labrador, Prince-Edward-Island, New Brunswick, and Nova Scotia were combined into the Atlantic Region; the three Prairie provinces (Manitoba, Saskatchewan, and Alberta) were also regrouped given the smaller sample sizes in these provinces. Moreover, due to a small number of observations under “other” gender identities, this group was excluded and gender was regarded as a binary variable (males and females). For similar reasons, the Indigenous status variable was regrouped into two categories, Indigenous and non-Indigenous, and the visible minority status variable was reported as either not a visible minority or part of a visible minority. It was not possible to obtain information on household income. Instead, education, dwelling ownership status, and household composition were used as proxy variables for socioeconomic status (SES). The former two indicators have also been used as valid SES measures in other studies [14]. Household composition is also used as a proxy for SES since it has been found that poverty rates change based on the type of household composition [15].

#### Health-related factors

Many health-related questions were included in the CCHS. For this study, we focused on questions regarding status of COVID-19 diagnosis, perceived health status, and having a regular healthcare provider as additional predictor variables.

#### Control variables

Region of residence, age group, and Indigenous status were used to adjust for differences in vaccination rollout plans and vaccination eligibility across the provinces. Therefore, any association observed between vaccination status and control variables should be interpreted with caution as differences in provincial vaccine eligibility could confound them.

### Statistical analysis

All analyses were conducted using SAS software version 9.4 of the SAS System for Windows.^1^ Descriptive statistics (frequencies and percentages) were computed to examine the distribution of vaccination status, vaccination intent, as well as sociodemographic factors in the population. To compare vaccination status among sociodemographic groups, the proportion of unvaccinated individuals was broken down by sociodemographic factors. Likewise, the proportion of those unlikely to get vaccinated by sociodemographic factor was also calculated to compare vaccination intent among different groups. The confidence interval of these proportions were then adjusted using the Wilson interval to account for the normal approximation of the binomial distribution [16]. For the final analysis, unadjusted (simple) and adjusted (multiple) logistic regression models were employed to examine associations between the response (vaccination status or intent) and predictor (sociodemographic and health-related) variables. The unadjusted models ran each of the predictor variables against each of the two outcomes, whereas the adjusted models included all predictor variables in the model.

To identify the sociodemographic determinants of non-vaccination and low vaccination intent, we examined the odds ratios (OR) generated by the logistic regression models. Specifically for vaccination status, we tested differences in the odds of being unvaccinated versus having at least one dose among sociodemographic groups. Similarly, for intent, we tested differences in the odds of being unlikely to get vaccinated versus being already vaccinated or likely to get vaccinated. A Tukey–Kramer adjustment was applied to the confidence intervals associated with the odds ratios to account for multiple comparisons.

Sampling weights were used to obtain accurate and representative estimates for the proportions and odds ratios, and their corresponding estimated variances were calculated using 1,000 bootstrap weights. All estimates were computed using a significance level of alpha = 0.05.

## Results

A total of 9,509 adult respondents from the 10 provinces were included in the analyses. Table 1 describes the sociodemographic distribution of the study sample. Of the five regions, British Columbia had the least number of respondents (9.1%) while Ontario had the most (30.6%). For gender, 55.7% of the sample were female, 44.1% were male and 0.2% had other or unknown gender identity. Sample proportions increased with increasing age groups from 8.1% in the 18–29 age group to 29.4% in the 70 and over. Almost two thirds of the sample had at least a post-secondary education (63.7%) and more than half (55.9%) were either married or living common-law. Lastly, 4.6% reported being Indigenous (i.e. identifying with at least one of the three Indigenous groups: First Nations, Métis, or Inuit) and 10.0% identified as being part of a visible minority group.

**Table 1:** Characteristics of survey respondents

Variables	Unweighted		Weighted
	Frequency (n)	Percent (%)	Percent (%)
Region of Residence			
Atlantic	1893	19.9	6.6
Quebec	1540	16.2	22.7
Ontario	2906	30.6	39.4
Prairies	2309	24.3	17.4
British Columbia	861	9.1	13.9
Gender			
Female	5295	55.7	50.7
Male	4197	44.1	49.3
Other or Unknown	17	0.2	-
Age Group			
18–29	769	8.1	18.2
30–39	1210	12.7	17.6
40–49	1150	12.1	16.7
50–59	1346	14.2	15.3
60–69	2242	23.6	17.1
70 +	2792	29.4	15.2
Level of Education			
Less than secondary	1263	13.3	9
Secondary	2129	22.4	22.1
Post-secondary	3144	33.1	31.4
University	2913	30.6	37.5
Unknown	60	0.6	-
Marital Status			
Married/Common law	5317	55.9	61.7
Divorced/Separated/Widowed	2236	23.5	12.1
Single	1945	20.5	26.2
Unknown	11	0.1	-
Indigenous Identity^a^			
Indigenous	433	4.6	3.2
Non-indigenous	8966	94.3	96.8
Unknown	110	1.2	-
Indigenous Identity			
First Nation	196	2.1	1.4
Metis	209	2.2	1.7
Inuit	F	F	F
Multiple	F	F	F
Non-indigenous	8966	94.3	96.9
Unknown	119	1.3	-
Visible Minority Status			
Visible minority	951	10	22.5
Not a visible minority	8426	88.6	77.5
Unknown	132	1.4	-
Immigration Status			
Non-Immigrant	7827	82.3	73.5
Immigrant	1465	15.4	24.3
Non-permanent resident	117	1.2	2.3
Unknown	100	1.1	-
Country of Birth			
Canada	7827	82.3	73.2
Other	1596	16.8	26.8
Unknown	86	0.9	-
COVID-19 Status			
Had COVID-19	181	1.9	3.6
Did not have COVID-19	9050	95.2	96.4
Unknown	278	2.9	-
Household Composition			
Unattached	3176	33.4	16
Couple	3286	34.6	29.1
Couple with children	2100	22.1	41.5
Lone with children	679	7.1	8.2
Other or Unknown	268	2.8	5.2
Employment Status			
Employed	4497	47.3	62.4
Unemployed	3483	36.6	29.5
Not in the labour force	1486	15.6	8.1
Unknown	43	0.5	-
Having a Regular Healthcare Provider			
Yes	8360	88	85.3
No	1129	11.9	14.7
Unknown	13	0.1	-
Self-Perceived Health			
Excellent, very good or good	8072	84.9	88.5
Fair or poor	1426	15	11.5
Unknown	11	0.1	-
Children under 12 in the household			
None	8159	85.8	79.8
1 or more	1350	14.2	20.2
Mother Tongue			
English or French	8394	88.2	81.9
Neither English or French	1018	10.7	18.1
Unknown	97	1	-
Dwelling Ownership Status			
Rent	1991	20.9	22.7
Own	7431	78.1	77.3
Unknown	87	0.9	-
Vaccination Status			
At least one dose	8151	85.7	88.7
Unvaccinated	1072	11.3	11.3
Unknown	286	3	-
Vaccination Intent			
Likely	494	5.2	6
Unlikely	553	5.8	5
Already vaccinated	8151	85.7	89
Unknown	311	3.3	-

^a^ Indigenous group includes First Nations, Metis, and/or Inuit

F Suppressed due to data quality concerns and/or confidentiality reasons

### Vaccination

According to this nationally representative sample, COVID-19 vaccination coverage in the ten Canadian provinces from June to September 2021 was 89% (Table 2). Overall, the proportion of unvaccinated did not differ by region. Differences across genders, visible minority status, mother tongue, and history of COVID-19 diagnosis were also not detected although vaccination rates improved with increasing age.

**Table 2:** Associations between sociodemographic factors and vaccination status: Odds of being unvaccinated vs. vaccinated

Predictors	Sample Size^a^	% Not vaccinated	Simple Logistic Regression		Multiple Logistic Regression^b^	
	n	% (95% CI^c^)	OR (95% CI^d^)	*P*-value	aOR (95% CI^d^)	*P*-value
Overall	9223	11 (10–13)				
Region of Residence^e^						
Atlantic	1812	10 (9–13)	1.1 (0.7–1.6)	0.996	1.3 (0.8–2.1)	0.493
Quebec	1513	10 (8–12)	Reference		Reference	
Ontario	2808	12 (10–14)	1.2 (0.8–1.9)	0.81	1.5 (0.9–2.5)	0.227
Prairies	2253	13 (11–15)	1.3 (0.8–2.1)	0.412	1.5 (0.9–2.6)	0.179
British Columbia	837	11 (8–14)	1.1 (0.6–1.9)	0.989	1.5 (0.8–2.7)	0.446
Age Group^e^						
18–29	752	17 (13–21)	**4.4 (2.6–7.4)**	** < 0.001**	**6.0 (2.5–14.7)**	** < 0.001**
30–39	1183	15 (12–18)	**3.8 (2.3–6.3)**	** < 0.001**	**5.9 (2.5–13.7)**	** < 0.001**
40–49	1139	13 (11–17)	**3.5 (2.1–5.7)**	** < 0.001**	**5.1 (2.2–11.9)**	** < 0.001**
50–59	1331	9 (8–12)	**2.3 (1.4–3.8)**	** < 0.001**	**3.2 (1.5–6.8)**	** < 0.001**
60–69	2193	7 (6–9)	**1.8 (1.1–2.9)**	**0.018**	1.8 (0.9–3.6)	0.114
70 +	2625	4 (3–6)	Reference		Reference	
Indigenous Identity^e^						
Indigenous	419	19 (13–26)^E^	**1.9 (1.2–2.9)**	**0.004**	1.4 (0.8–2.2)	0.214
Non-Indigenous	8697	11 (10–12)	Reference		Reference	
Gender						
Male	4009	11 (9–12)	1.2 (0.9–1.4)	0.185	1.1 (0.9–1.4)	0.36
Female	5199	12 (10–14)	Reference		Reference	
Level of Education						
Less than secondary	1181	16 (12–20)	**2.5 (1.5–4.0)**	** < 0.001**	**3.5 (2.1–6.1)**	** < 0.001**
Secondary	2063	13 (11–16)	**2.0 (1.4–3.1)**	** < 0.001**	**2.4 (1.5–3.7)**	** < 0.001**
Post-secondary	3070	14 (12–16)	**2.1 (1.4–3.0)**	** < 0.001**	**2.5 (1.6–3.7)**	** < 0.001**
University	2856	7 (6–9)	Reference		Reference	
Employment Status						
Employed	4443	12 (10–14)	Reference		Reference	
Unemployed	3371	11 (10–13)	1.0 (0.7–1.3)	0.901	1.2 (0.8–1.6)	0.618
Not in the labour force	1367	4 (3–6)^E^	**0.3 (0.2–0.5)**	** < 0.001**	0.9 (0.4–1.8)	0.892
Marital Status						
Divorced/Separated/Widowed	2189	13 (11–16)	**1.5 (1.1–2.2)**	**0.011**	1.6 (0.8–3.2)	0.297
Single	1901	16 (13–19)	**1.8 (1.4–2.5)**	** < 0.001**	1.2 (0.7–2.2)	0.634
Married/Common law	5122	9 (8–10)	Reference		Reference	
Immigration Status						
Non-Immigrant	7604	11 (10–12)	Reference		Reference	
Non-Permanent Resident	112	27 (16–41)^E^	**3.1 (1.3–7.5)**	**0.009**	2.2 (0.8–6.2)	0.182
Immigrant	1411	11 (9–14)	1.1 (0.8–1.5)	0.806	1.5 (0.9–2.3)	0.147
Visible Minority Status						
Not a visible minority	8170	11 (10–12)	Reference		Reference	
Visible minority	923	12 (9–15)	1.1 (0.8–1.5)	0.45	0.7 (0.4–1.2)	0.178
Mother Tongue						
English or French	8160	11 (10–12)	Reference		Reference	
Neither English or French	968	13 (10–17)	0.9 (0.5–1.5)	0.45	1.2 (0.7–1.8)	0.512
Children under 12 in the household						
1 or more	1323	15 (12–17)	**1.5 (1.2–1.8)**	**0.001**	**1.6 (1.1–2.4)**	**0.013**
None	7900	10 (9–12)	Reference		Reference	
Household Composition						
Unattached	3147	13 (11–16)	**1.8 (1.2–2.7)**	**0.001**	1.3 (0.6–2.7)	0.927
Couple with children	2052	11 (9–13)	1.4 (0.9–2.1)	0.14	0.6 (0.3–1.1)	0.207
Lone with children	644	20 (15–25)	**2.9 (1.7–4.8)**	** < 0.001**	1.1 (0.5–2.8)	0.996
Couple	3119	8 (6–10)	Reference		Reference	
Other, Unknown	261	14 (9–22)^E^	1.3 (0.9–1.7)	0.135	0.8 (0.3–2.3)	0.974
Dwelling Ownership Status						
Rent	1949	15 (13–18)	**1.7 (1.3–2.1)**	** < 0.001**	1.0 (0.7–1.3)	0.829
Own	7190	10 (9–11)	Reference		Reference	
Having a Regular Healthcare Provider						
No	1105	18 (14–22)	**1.9 (1.4–2.5)**	** < 0.001**	**1.6 (1.1–2.2)**	**0.006**
Yes	8098	10 (9–11)	Reference		Reference	
Self-Perceived Health						
Fair or poor	1310	16 (13–20)	**1.6 (1.2–2.1)**	**0.001**	**1.8 (1.3–2.4)**	** < 0.001**
Excellent, very good or good	7903	11 (10–12)	Reference		Reference	
COVID-19 Status						
Did not have COVID-19	9029	11 (10–12)	0.7 (0.4–1.4)	0.342	0.7 (0.4–1.5)	0.37
Had COVID-19	181	14 (8–24)^E^	Reference		Reference	

**Bold** values indicate significant odds ratios after adjustment at the 5% level

^a^ Sample sizes for proportions and simple logistic regression models do not always add up to the total *n* = 9,509 due to missing values in predictor and outcome variables

^b^ Sample size for the multiple logistic regression is *n* = 8,908

^c^ Wilson score interval for binomial proportions

^d^ 95% confidence intervals for odds ratios (OR) were adjusted using the Tukey–Kramer method for multiple comparisons

^e^ Covariates to control for provincial differences in vaccination rollout plans and vaccination eligibility

^E^ Estimate is of marginal quality, use with caution

**Fig. 1:**
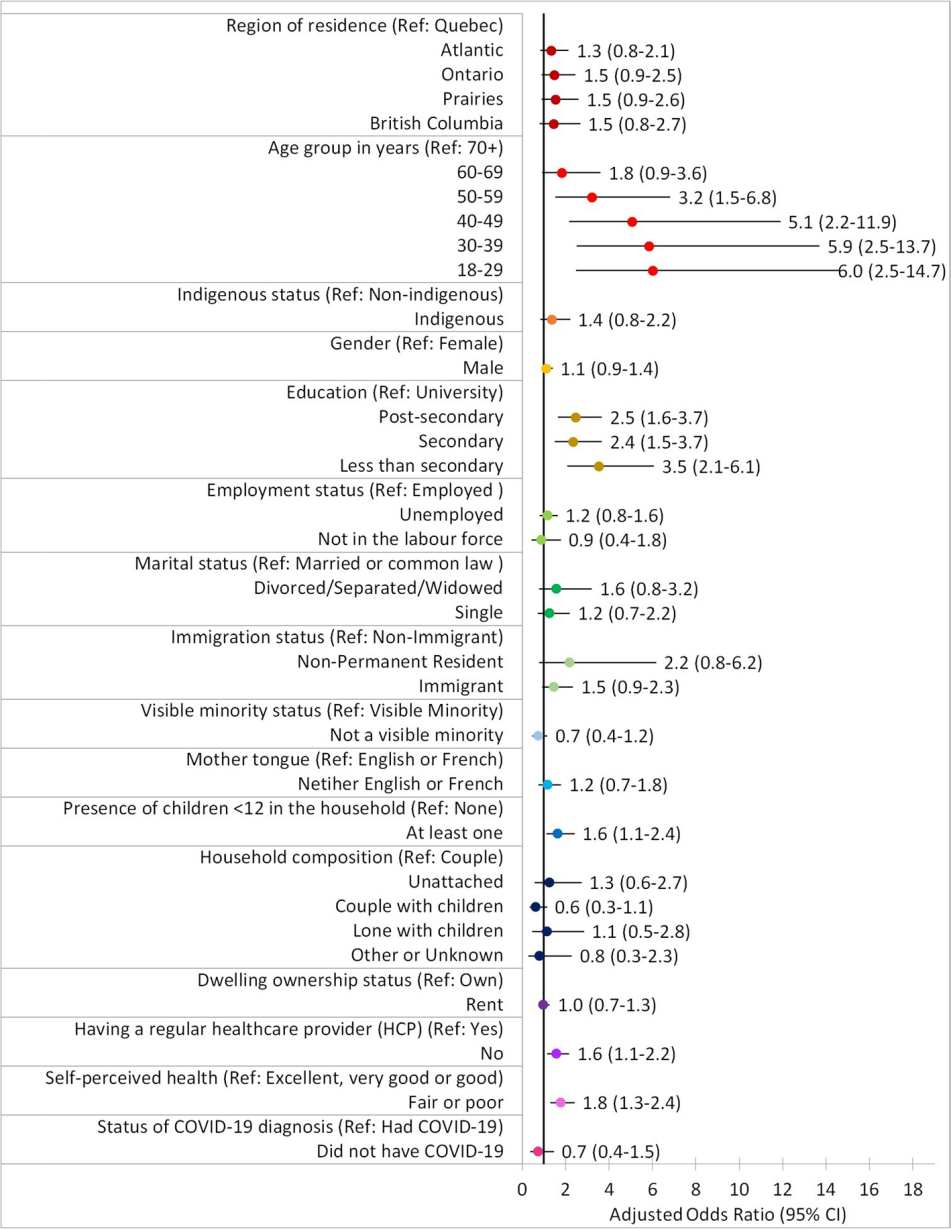
Adjusted odds ratios: Being unvaccinated versus vaccinated by sociodemographic and health factors

After adjusting for covariates, vaccination status was associated with level of education, presence of children under 12 years old in the household, having a regular healthcare provider, and self-perceived health (Table 2 and Fig. 1). Adults with education below university were more likely to be unvaccinated than university graduates, with adjusted odds ratios (aOR) ranging from 2.4 (95% CI 1.5–3.7) to 3.5 (95% CI 2.1–6.1). Those living with at least one child under 12 years old were also more likely to be unvaccinated than those living without children (aOR 1.6, 95% CI 1.1–2.4). The risk of being unvaccinated was also higher in those without a regular healthcare provider compared to those who had one (aOR 1.6, 95% CI 1.1–2.2) and in those who perceived their health as fair or poor compared to those who perceived it as excellent, very good or good (aOR 1.8, 95% CI 1.3–2.4). As expected, due to the vaccine rollout by descending age group, vaccination decreased significantly with decreasing age.

### Intent to get vaccinated

**Fig. 2:**
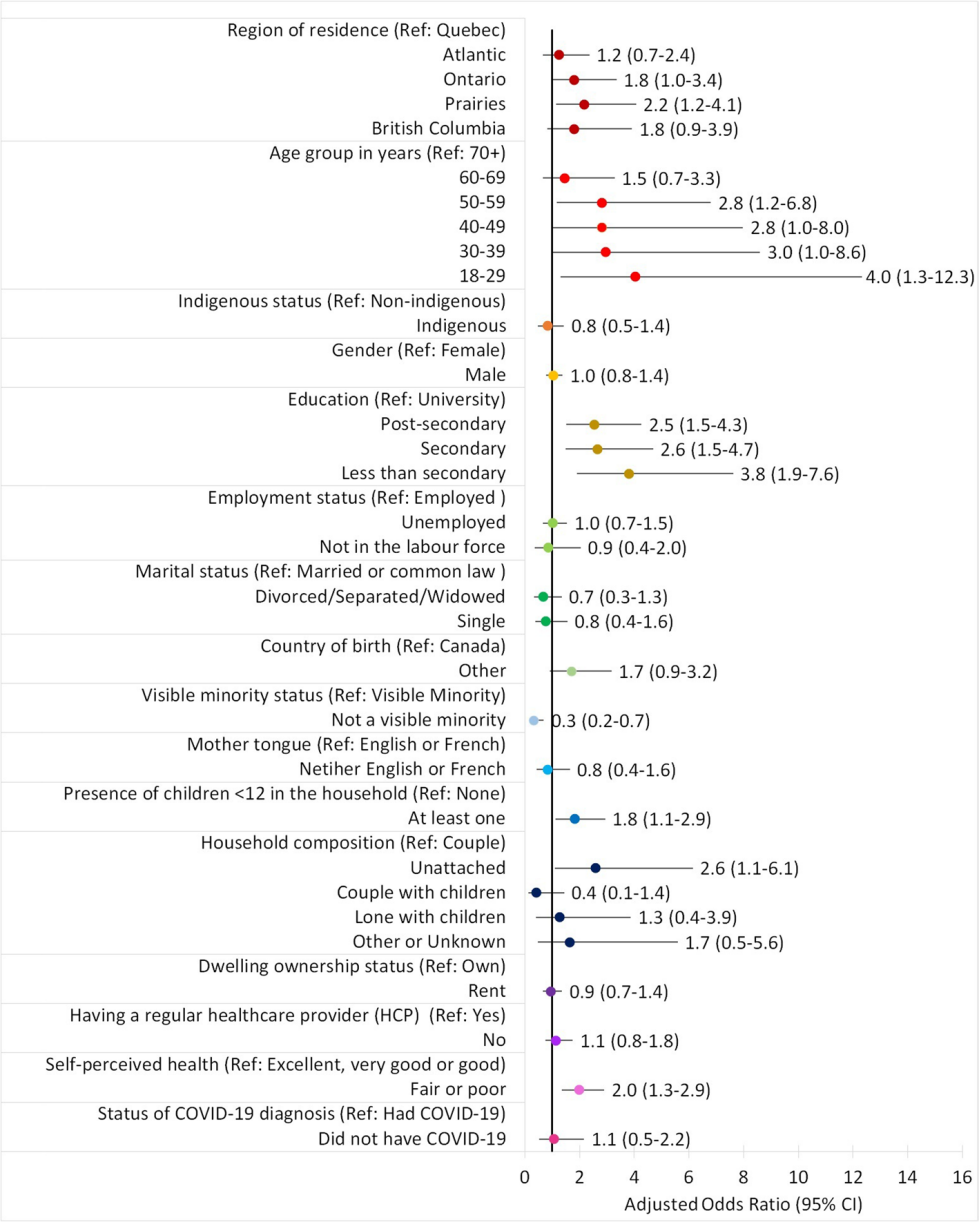
Adjusted odds ratios: Unlikely to get vaccinated versus likely or vaccinated by sociodemographic factors

Only 5% of the population did not intend to get vaccinated (Table 3). The proportion of those unlikely to get vaccinated were slightly higher in the Prairies compared to the other regions. Vaccination intent did not differ across Indigenous identity, gender, employment status, marital status, country of birth, mother tongue, dwelling ownership status, having a regular healthcare provider, or status of COVID-19 diagnosis. Based on the adjusted model, vaccination intent was associated with region of residence, age, level of education, visible minority status, presence of children under 12 years old in the household, household composition, and self-perceived health (Table 3 and Fig. 2). The risk of being unlikely to get vaccinated was greater in the Prairies compared to the risk in the province of Quebec (aOR 2.2, 95% CI 1.2–4.1). Additional comparisons among the regions also showed that risks were greater in the Prairies than in the Atlantic region. Greater risks were also observed in those less than 40 years old and between 50 to 59 compared to those 70 and over (aOR between 2.8, 95% CI 1.2–6.8 and 4.0, 95% CI 1.3–12.3). The intent not to get vaccinated was more frequent in individuals with no university degree than in university graduates (aOR between 2.5, 95% CI 1.5–4.3 and 3.8, 95% CI 1.9–7.6). The intent not to get vaccinated was also more frequent in those living with at least one child under 12 years old than in those with no children (aOR 1.8, 95% CI 1.1–2.9), more frequent in unattached individuals than in those living in a couple (aOR 2.6, 95% CI 1.1–6.1), and more frequent in those who perceived their health as fair or poor than in those perceiving it as excellent, very good or good (aOR 2.0, 95% CI 1.3–2.9). However, decreased risks (aOR 0.3, 95% CI 0.2–0.7) of being unlikely to get vaccinated were observed in those who are part of a visible minority group compared to those who are not a visible minority.

**Table 3:** Sociodemographic indicators of intent: Odds of being unlikely to get vaccinated vs. likely or vaccinated

	Sample Size^a^	Unlikely to get vaccinated	Simple Logistic Regression		Multiple Logistic Regression^b^	
Predictors	n	% (95% CI^c^)	OR (95% CI^d^)	*P*-value	aOR (95% CI^d^)	*P*-value
Overall	9198	5 (4–6)				
Region of Residence^e^						
Atlantic	1804	4 (3–6)	1.1 (0.6–2.0)	0.988	1.2 (0.7–2.4)	0.885
Quebec	1511	4 (3–5)^E^	Reference		Reference	
Ontario	2796	5 (4–7)	1.3 (0.7–2.4)	0.686	1.8 (1.0–3.4)	0.083
Prairies	2251	7 (5–8)	1.8 (1.0–3.2)	0.064	**2.2 (1.2–4.1)**	**0.007**
British Columbia	836	5 (3–7)^E^	1.2 (0.6–2.6)	0.964	1.8 (0.9–3.9)	0.239
Age Group^e^						
18–29	749	6 (4–8)^E^	1.9 (0.9–3.9)	0.122	**4.0 (1.3–12.3)**	**0.005**
30–39	1178	6 (4–8)^E^	1.9 (1.0–3.6)	0.054	**3.0 (1.0–8.6)**	**0.043**
40–49	1131	6 (4–8)^E^	1.9 (1.0–3.6)	0.061	2.8 (1.0–8.0)	0.052
50–59	1326	6 (4–7)	**1.9 (1.0–3.4)**	**0.036**	**2.8 (1.2–6.8)**	**0.011**
60–69	2192	4 (3–6)	1.4 (0.8–2.6)	0.512	1.5 (0.7–3.3)	0.763
70 +	2622	3 (2–4)	Reference		Reference	
Indigenous Identity^e^						
Indigenous	418	7 (4–11)^E^	1.5 (0.9–2.4)	0.115	0.8 (0.5–1.4)	0.497
Non-indigenous	8674	5 (4–6)	Reference		Reference	
Gender						
Male	3996	5 (4–6)	1.1 (0.8–1.5)	0.456	1.0 (0.8–1.4)	0.79
Female	5187	5 (4–6)	Reference		Reference	
Level of Education						
Less than secondary	1177	8 (6–11)^E^	**3.3 (1.8–6.0)**	** < 0.001**	**3.8 (1.9–7.6)**	** < 0.001**
Secondary	2055	6 (5–8)	**2.5 (1.5–4.4)**	** < 0.001**	**2.6 (1.5–4.7)**	** < 0.001**
Post-secondary	3064	6 (5–8)	**2.6 (1.6–4.2)**	** < 0.001**	**2.5 (1.5–4.3)**	** < 0.001**
University	2849	3 (2–3)^E^	Reference		Reference	
Employment Status						
Employed	4427	5 (4–6)	Reference		Reference	
Unemployed	3363	5 (4–6)	1.0 (0.7–1.4)	0.944	1.0 (0.7–1.5)	0.999
Not in the labour force	1367	3 (2–5)^E^	0.6 (0.4–1.0)	0.063	0.9 (0.4–2.0)	0.913
Marital Status						
Divorced/Separated/Widowed	2185	6 (5–8)	1.5 (1.0–2.2)	0.065	0.7 (0.3–1.3)	0.38
Single	1887	6 (4–8)^E^	1.4 (0.9–2.1)	0.132	0.8 (0.4–1.6)	0.664
Married/Common law	5115	4 (4–5)	Reference		Reference	
Country of Birth						
Canada	7586	5 (5–6)	Reference		Reference	
Other	1529	4 (3–6)^E^	0.8 (0.5–1.1)	0.131	1.7 (0.9–3.2)	0.09
Visible Minority Status						
Not a visible minority	8151	6 (5–6)	Reference		Reference	
Visible minority	920	3 (2–4)^E^	**0.5 (0.3–0.8)**	**0.003**	**0.3 (0.2–0.7)**	**0.004**
Mother Tongue						
English or French	8142	5 (4–6)	Reference		Reference	
Neither English or French	961	3 (2–5)^E^	0.6 (0.4–1.0)	0.055	0.8 (0.4–1.6)	0.593
Children under 12 in the household						
1 or more	1319	6 (5–8)	1.2 (0.9–1.8)	0.161	**1.8 (1.1–2.9)**	**0.016**
None	7879	5 (4–6)	Reference		Reference	
Household Composition						
Unattached	3134	7 (6–9)	**2.1 (1.3–3.3)**	** < 0.001**	**2.6 (1.1–6.1)**	**0.022**
Couple	3116	4 (3–5)	Reference		Reference	
Couple with children	2047	4 (3–6)	1.2 (0.7–2.0)	0.909	0.4 (0.1–1.4)	0.313
Lone with children	640	6 (4–10)^E^	1.8 (0.9–3.5)	0.132	1.3 (0.4–3.9)	0.977
Other or Unknown	261	7 (4–13)^E^	2.0 (0.7–5.5)	0.312	1.7 (0.5–5.6)	0.796
Dwelling Ownership Status						
Rent	1935	6 (5–8)	1.3 (1.0–1.8)	0.095	0.9 (0.7–1.4)	0.763
Own	7179	5 (4–5)	Reference		Reference	
Having a Regular Healthcare Provider						
No	1101	6 (4–8)^E^	1.2 (0.8–1.7)	0.291	1.1 (0.8–1.8)	0.529
Yes	8077	5 (4–6)	Reference		Reference	
Self-Perceived Health						
Fair or poor	1305	9 (6–12)^E^	**2.0 (1.4–2.9)**	** < 0.001**	**2.0 (1.3–2.9)**	** < 0.001**
Excellent, very good or good	7884	5 (4–5)	Reference		Reference	
COVID-19 Status						
Did not have COVID-19	9004	5 (4–6)	1.2 (0.6–2.4)	0.52	1.1 (0.5–2.2)	0.865
Had COVID-19	181	F	Reference		Reference	

**Bold** values indicate significant odds ratios after adjustment at the 5% level

^a^ Sample sizes for proportions and simple logistic regression models do not always add up to the total *n* = 9,509 due to missing values in predictor and outcome variables

^b^ Sample size for the multiple logistic regression is *n* = 8,905

^c^ Wilson score interval for binomial proportions

^d^ 95% confidence intervals for odds ratios (OR) were adjusted using the Tukey–Kramer method for multiple comparisons

^e^ Covariates to control for provincial differences in vaccination rollout plans and vaccination eligibility

^E^ Estimate is of marginal quality, use with caution

F Estimate is suppressed due to data quality concerns

## Discussion

### COVID-19 vaccination

In this study, vaccination coverage among Canadian adults was high, with 89% having received at least one dose of a COVID-19 vaccine. Only 11% of the population were unvaccinated. This coverage is somewhat higher than what was reported by the Canadian COVID-19 vaccination coverage surveillance system where by beginning of September 2021, 84% of those 18 and older received at least one dose, which may be explained by differences between the CCHS respondents and the general population [17]. Among all the sociodemographic factors included in the vaccination status model, age, level of education, presence of children under 12 years old in the household, having a healthcare provider and self-perceived health were identified as significant determinants of COVID-19 vaccine uptake.

The proportion of unvaccinated individuals decreased with increasing age ranging from 17% in 18–29 years old to 4% in those aged 70 years or over. This can be partially explained by the vaccination rollout, as the elderly population was eligible to get vaccinated earlier in the campaign [18]. Analogously, some studies highlighted that risk perceptions toward COVID-19 differ by age [19, 20]. Given that the severity and mortality of COVID-19 increases with age [21], older adults might feel more at risk, thus more motivated to be vaccinated. In fact, increased risk perceptions of COVID-19 have also been shown to be a strong predictor of COVID-19 vaccine acceptance [22].

In this study, only one of the three proxy variables for SES, namely education, was significantly associated with vaccination status. Individuals with less than university education had a higher risk of being unvaccinated than university graduates. Similarly, a recent study in the US looked at patterns in COVID-19 vaccination coverage among adults and reported that vaccination uptake was lower among adults with low educational attainment, which is consistent with our results [23]. Education is proven to be associated with greater engagement in pro-health behaviors which could be seen as a factor in favor of vaccination [24].

According to the CCHS results, individuals who lived with at least one child under 12 years old had a higher risk of being unvaccinated. One might postulate that this association is driven by age since individuals living with young children are, for the vast majority, younger adults. However, it should be noted that this association is still significant when adjusted for age groups. A study in the US found similar results where the presence of children was negatively associated with COVID-19 vaccination [23]. As postulated by Bell et al., this may be related to access barriers to vaccination; parents of younger children may face obstacles to schedule and attend vaccination appointments due to competing priorities [25].

The risk of being unvaccinated was also significantly higher in those who had fair or poor self-perceived health compared to those with excellent, very good or good self-perceived health (aOR 1.8). This could be explained by the fact that individuals with poor health had less intent to get vaccinated, and therefore the coverage was lower. A study in the U.S found that those with underlying medical conditions and BMI > 40 were not more willing to get vaccinated than those without these risk factors [26]. This could be associated with different perceptions on vaccine safety, side effects and effectiveness among those with poor health. One study demonstrated that people cared more about the vaccine’s health risk than its effectiveness [27]. Therefore, those with poor health may be more concerned with health risks associated with getting vaccinated than being immunized against COVID-19. On top of that, reduced COVID-19 mortality risk following immunization may in part explain the current finding as it suggests substantial “healthy vaccinee effects” which refers to a situation when vaccinated individuals tend to be healthier than unvaccinated individuals [28]. This pattern is the opposite of what was observed for influenza vaccination in Canada in previous cycles of the same survey (CCHS) where excellent self-perceived health was associated with non-vaccination among adults aged 18 to 64 years with a chronic medical condition and in adults aged 65 years and older [29].

Finally, having a regular healthcare provider was positively associated with COVID-19 vaccine uptake. It is conceivable that individuals with a regular healthcare provider may have easier access to health-related resources and may be more willing to get vaccinated in order to protect themselves from the disease. Additionally, multiple studies have demonstrated that those who are hesitant to get a COVID-19 vaccine are concerned about the safety of the vaccines and the risks and side effects attached to it [22, 30–33]. Considering healthcare providers as being one of the trusted sources of information on vaccination, they may help to soothe the fear and concerns over COVID-19 vaccines, reassure their patients on the safety and effectiveness of the vaccines, and promote vaccination during consultations, which can therefore improve vaccine uptake [8, 34].

### COVID-19 vaccination intent

The proportion of the population who did not intend to get vaccinated against COVID-19 was as low as 5%. When adjusting for all other predictor variables, lower vaccination intent was significantly associated with region, younger age, lower educational attainment, not being part of a visible minority group, presence of children under 12 years in the household, unattached individuals and poor self-perceived health.

Vaccination intent differed between the Canadians provinces. Individuals living in the Prairies had higher risks of not intending to get vaccinated compared to other provinces. In the same vein, another Canadian study also found that these three provinces had higher proportions of individuals who did not intend to get vaccinated [7]. However, once adjusted for other sociodemographic factors, these differences were not significant despite individuals from Alberta having higher predicted probability of not intending to get vaccinated [7].

Adults younger than 40 years old and between 50 to 59 had lower COVID-19 vaccination intent than those aged 70 or over. Other Canadians studies had similar results where individuals below 60 years of age demonstrated lower intent to get vaccinated [5, 7]. A systematic review of 45 studies conducted in various countries hypothesized that older individuals have a greater sense of responsibility and accountability for themselves and their surroundings compared to the younger population which may also explain why older individuals were more likely to be vaccinated [35].

Following the pattern of vaccination status, the intent not to get vaccinated was more frequent in individuals with no university degree than in those who held such a degree. This is in agreement with another Canadian study that reported having a university education level as one of the strongest predictors of COVID-19 vaccine intention [22]. Education level plays an important role in vaccination acceptance as it highly correlates with belief in COVID-19 vaccine safety [36]. According to Kricorian et al., individuals who believed COVID-19 vaccines to be unsafe were likely to have difficulty understanding scientific information, higher mistrust in scientific research, and not to follow scientific recommendations. This could contribute to the lower intent of receiving the vaccine.

Moreover, visible minorities overall were found to be more eager to get vaccinated than the rest of the population. A major caveat to this finding was that it applies to visible minorities as a whole, as the number of participants from these groups in this study was not sufficient to analyse them separately. Supporting evidence from a US study indicates that some visible minority groups such as Asians and Hispanics are less likely to have vaccine hesitancy than Whites across all hesitancy measures [37]. Nevertheless, according to other studies in other countries, higher vaccine hesitancy was observed in most minority ethnic groups compared to the White British or Irish group; and identifying as Black or African American was associated with lower vaccination likelihood as opposed to identifying as White [38, 39]. The association between visible minority status and vaccination intent observed in the current study may in part be explained by the multi-ethnic characteristic of the Canadian healthcare workers. In Canada, visible minorities account for approximately one third of nurse aides, orderlies and patient service associates, with higher proportions of Black, Filipino and South Asian workers in these occupations [40]. In addition to having been prioritized for vaccination, being at increased risk of COVID-19 infection and transmission may contribute to healthcare workers increased willingness to get vaccinated. Further exploration is essential to better understand the association between the various visible minority groups and COVID-19 vaccination intent.

Presence of children under 12 years old in the household was negatively associated with COVID-19 vaccination intent (aOR 1.8). A Canadian study on predictors of vaccine hesitancy for COVID-19 public health messaging implications revealed that having more than three children in the family is a strong determinant of immunization noncompliance [41]. This finding is also consistent with other research where presence of children in the household increased the odds of vaccine hesitancy [37, 42]. It could seem counterintuitive given that having multiple children ought to encourage parents to vaccinate in order to prevent cross-infection within the household. Nonetheless, relations between family size and vaccination intent may be explained through other socioeconomic factors.

Additionally, unattached individuals had lower COVID-19 vaccination intent than coupled individuals. A US nationwide study on predictors of intention to vaccinate against COVID-19 also demonstrated that having a spouse or partner was associated with higher anticipated likelihood of vaccination [43]. Unattached individuals might not have as much collective family responsibilities as married individuals and those with children, which could explain their lower vaccination intent [35].

Lastly, the current study showed that individuals with fair or poor self-perceived health had lower intention to vaccinate against COVID-19 than those with excellent, very good, good self-perceived health. This is in agreement with a study on COVID-19 vaccine hesitancy associated factors in Saskatchewan where individuals with very good or excellent health status were more likely to vaccinate than those with poor of fair health status [6]. However, more research is warranted to examine the association between self-perceived health and COVID-19 vaccination intent, especially since many perceived that aspects of their overall health had deteriorated during the pandemic [44].

In the present study, some sociodemographic factors such as gender, immigration status, Indigenous identity, and employment status were not significantly associated with COVID-19 vaccination uptake or intent. However, previous studies conducted in Canada showed that being a male was positively associated with vaccination intent [22, 45]. Although no difference in uptake for Indigenous status was observed in the adjusted model, Indigenous groups might still differ from non-Indigenous. It should be noted that statistical non-significance is not proof of absence of an association. Sometimes, the non-significant result is due to lack of power rather than lack of effect; the sample size of Indigenous respondents might be too small to provide sufficient power to detect an association. This can also be true for small groups of other variables such as immigration status or COVID-19 status. Other Canadian studies with various sample sizes of Indigenous respondents and somewhat different target populations found that Indigenous groups and individuals born outside of Canada had lower odds of getting vaccinated [5–7].

Given the paucity of studies assessing inequalities in COVID-19 vaccination uptake and intent, further work is needed for a deeper understanding of the contributing factors in the Canadian context.

Factors other than sociodemographic can also play a role in vaccination uptake and intent. Health inequities, vaccine hesitancy as well as knowledge, attitudes and beliefs (KABs) are among the multitude of factors that can have an impact. Although some KABs have been associated with sociodemographic characteristics, including only sociodemographic variables in the models might not provide a comprehensive picture. Unfortunately, information on KABs was not collected in the CCHS. Nonetheless, the assessment of sociodemographic factors can inform interventions by identifying target groups. Some people do not intend to get vaccinated due to concerns about the safety and effectiveness of the vaccine [22]. The novelty of COVID-19 vaccines could also play a role in Canadians’ intent to get vaccinated, as well as their lack of knowledge about vaccination [22, 45].

### Strengths and limitations

As with any large scale survey, the CCHS has several strengths and limitations that must be carefully considered when interpreting the results. A major strength of the survey was the sufficiently large sample size to allow for analysis by several sociodemographic and health-related factors. Additionally, given the complex survey design and the use of survey weights, the findings are nationally representative and allows us to make inferences to the Canadian population. This study can also be a catalyst to potential additional works to examine hypotheses on changes of vaccination status and intent over time, on intent at the provincial level, and on the impact of additional sociodemographic indicators such as household income and rural/urban living area. Most importantly, this study is one of few that examine vaccination status and intent at the national level in Canada, contributing to the growing body of research on COVID-19 vaccine acceptance or hesitancy.

Some study limitations need to be acknowledged. The CCHS shares the usual limitations of surveys based on self-reporting which may be subject to recall bias given that the data was collected more than 7 months after the beginning of COVID-19 vaccination. However, recall bias is less likely to occur in the present study due to high media coverage surrounding the COVID-19 vaccination campaign and the proof of vaccination credentials issued by many jurisdictions across Canada. There are also some limitations to collecting data only through telephone interviews [46]. As a result of the COVID-19 pandemic, no computer-assisted personal interviewing (CAPI) was conducted in 2021; only CATI was used to collect data. Consequently, CATI is limited by the fact that participants have the possibility to not answer the phone whereas they are a lot less comfortable refusing an interview when they are facing the interviewer in person often resulting in lower response rates for CATI compared to CAPI. Indeed, the CCHS response rates significantly decreased in 2021. As was done for previous CCHS cycles, survey weights were adjusted to minimize any potential bias that could arise from survey non-response; non-response adjustments and calibration using available auxiliary information were applied. Despite these rigorous adjustments and validations, the higher non-response rate increases the risk of a remaining bias and increases the magnitude with which such a bias could impact estimates produced using the survey data. Moreover, selection bias cannot be ruled out since individuals with greater interest in the topic could be more likely to respond to the survey. In addition, as with all surveys, the social desirability bias needs to be considered.

In addition, the small number of observations among visible minority groups prohibited a more detailed breakdown of the visible minority status variable by individual visible minority group. This may explain why our finding on visible minority is inconsistent with other studies conducted in Canada or elsewhere. As with many other Statistics Canada surveys, the CCHS excluded First Nations on-reserve communities. Moreover, for the Indigenous status variable, the small number of observations did not allow a further analysis broken down by First Nation, Métis and Inuit. Future research should strive to include a sufficient number of visible minority and Indigenous participants to allow more detailed analyses of intent to get vaccinated and vaccination coverage in these populations. Continued collection would allow for data pooling to increase the sample size and further explore sub-populations.

## Conclusion

Overall, a vast majority of the Canadian population was either vaccinated with at least one dose or likely to receive a COVID-19 vaccine. In this study, after adjustment for covariates, lower vaccination uptake was associated with younger age, lower educational attainment, presence of children in the household, not having a regular health care provider, and fair or poor self-perceived health. Furthermore, lower COVID-19 vaccination intent was associated with residing in the Prairies region, younger age, lower level of education, presence of young children in the household, fair or poor self-perceived health, not being part of a visible minority group and unattached individuals. Ongoing monitoring of inequalities in COVID-19 vaccination uptake and intent is needed to precisely identify vaccination barriers among partially vaccinated and unvaccinated populations. Addressing these barriers with better targeted interventions and promotion strategies is the key to achieve higher coverage rates and to protect all Canadians against the disease.

## Data Availability

The dataset analyzed during the current study is available in the Statistics Canada Research Data Centres, https://www.statcan.gc.ca/en/microdata/data-centres.

## Abbreviations

aOR: Adjusted odds ratio
CATI: Computer-assisted telephone interviewing
CCHS: Canadian Community Health Survey
CI: Confidence interval
COVID-19: Coronavirus disease of 2019
HCP: Healthcare provider
KABs: Knowledge, attitudes and beliefs
OR: Odds ratio
Ref: Reference
SAS: Statistical Analysis System
SES: Socioeconomic status
US: United States

## Footnotes

1 Copyright © 2017 SAS Institute Inc. SAS and all other SAS Institute Inc. product or service names are registered trademarks or trademarks of SAS Institute Inc., Cary, NC, USA.
